# How often are imaging studies for thoracic aortic aneurysm necessary?

**DOI:** 10.1007/s12471-021-01641-z

**Published:** 2021-11-11

**Authors:** M. J. Schuuring, J. J. Piek

**Affiliations:** Department of Cardiology, Amsterdam University Medical Centres—location AMC, University of Amsterdam, Amsterdam, The Netherlands

In recent years imaging studies have increased in number and complexity [1, 2]. Consequently, imaging laboratories face the ongoing, growing challenge of processing all of the clips and images they obtain daily. The increasing workload in all imaging laboratories as well as the varying image quality make thorough and timely interpretation challenging. Whereas an appropriate indication makes the request for an imaging study relevant, inappropriate indications may lead to overinvestigation and consequently overloading, leading to technicians dropping out owing to being both psychologically and physically overburdened.

A substantial proportion of imaging studies are requested to evaluate aortic diameters. Thoracic aorta aneurysms (TAAs) have a higher risk of type A aortic dissection, which is an emergency situation that needs prompt surgery to avert morbidity and mortality. The risk of a TAA dissection is known to correlate with the maximal aortic diameters. Therefore, frequent surveillance of the aortic diameters with an imaging modality is recommended. The guidelines of the European Society of Cardiology and American College of Cardiology describe clear indications for surgical procedures. However, the frequency of follow-up studies of these aortic diameters is insufficiently described in the guidelines because the optimal surveillance interval is unknown [3–5].

Adriaans et al. performed a retrospective single-centre study of their clinical database with patients who were referred for TAA evaluation at Maastricht University Medical Centre between January 1999 and August 2019 [6]. Interestingly, both patients with a bicuspid and tricuspid aortic valve were studied. Exclusion criteria comprised diagnosis or suspicion of connective tissue disease and prior aortic or valvular surgery. Aortic diameter growth rates were calculated from the differences between the first and last investigations. The diagnostic accuracy of follow-up protocols was calculated as the percentage of patients requiring prophylactic surgery in whom timely identification would have occurred. The authors concluded that patients with TAAs but no connective tissue disease have an aneurysm growth rate that is lower than those previously reported. The mean growth rate in their study was 0.2 ± 0.4 mm/year. Interestingly, 41% of all TAAs did not grow over time. Conversely, a bicuspid aortic valve was not associated with more rapid aortic growth. Female sex and aortic regurgitation predisposed to more rapid aortic growth. Therefore, the authors suggest that a lower imaging frequency might be a good alternative approach in patients without connective tissue diseases. Yearly imaging did not lead to changes in the management of small aneurysms. Finally, Adriaans et al. propose triennial imaging follow-up for ascending aortic aneurysms with diameters between 40 and 49 mm.

There are, however, limitations that need to be addressed. First, this is a single-centre study with a retrospective design. It is relatively easy to see in retrospect in which patients no aneurysm growth has occurred, but it is much more difficult to predict which aneurysms will not grow in the future. Second, it is unknown how certain it is that there is no genetic underlying condition. Third, only ascending aortic aneurysms were studied. Fourth, growth rates were assessed with a suspected linear growth pattern only. Finally, inter- and intra-observer variability and inconsistency between various imaging modalities may have introduced inaccuracies.

In 2019, assessment of aortic diameters and their correlation with prognosis were prioritised among the top 11 research questions by the Netherlands Society of Cardiology [7]. Therefore, the study by Adriaans et al. is important, as it has a relevant impact on daily clinical practice. Now it is time to determine ‘the right care at the right place’ with a prospective multicentre study to verify the results reported by Adriaans et al. where, as in Maastricht, collaboration between cardiologists and radiologists is to be recommended. This will make an important contribution to international guidelines, reduce the workload in imaging laboratories and, most importantly, improve patient care.
